# Regular, Intense Exercise Training as a Healthy Aging Lifestyle Strategy: Preventing DNA Damage, Telomere Shortening and Adverse DNA Methylation Changes Over a Lifetime

**DOI:** 10.3389/fgene.2021.652497

**Published:** 2021-08-06

**Authors:** Maha Sellami, Nicola Bragazzi, Mohammad Shoaib Prince, Joshua Denham, Mohamed Elrayess

**Affiliations:** ^1^Physical Education Department (PE), College of Education (CEdu), Qatar University, Doha, Qatar; ^2^Department of Health Sciences (DISSAL), Postgraduate School of Public Health, University of Genoa, Genoa, Italy; ^3^Division of Sports and Wellness, Department of Students Affairs, College of North Atlantic Qatar (CNAQ), Doha, Qatar; ^4^School of Health and Biomedical Sciences, RMIT University, Melbourne, VIC, Australia; ^5^Biomedical Center, Qatar University, Doha, Qatar

**Keywords:** physical activity, epigenetics, telomerase, gene, oxidative stress, epigenetic clock, skeletal muscle

## Abstract

Exercise training is one of the few therapeutic interventions that improves health span by delaying the onset of age-related diseases and preventing early death. The length of telomeres, the 5′-TTAGGG^*n*^-3′ tandem repeats at the ends of mammalian chromosomes, is one of the main indicators of biological age. Telomeres undergo shortening with each cellular division. This subsequently leads to alterations in the expression of several genes that encode vital proteins with critical functions in many tissues throughout the body, and ultimately impacts cardiovascular, immune and muscle physiology. The sub-telomeric DNA is comprised of heavily methylated, heterochromatin. Methylation and histone acetylation are two of the most well-studied examples of the epigenetic modifications that occur on histone proteins. DNA methylation is the type of epigenetic modification that alters gene expression without modifying gene sequence. Although diet, genetic predisposition and a healthy lifestyle seem to alter DNA methylation and telomere length (TL), recent evidence suggests that training status or physical fitness are some of the major factors that control DNA structural modifications. In fact, TL is positively associated with cardiorespiratory fitness, physical activity level (sedentary, active, moderately trained, or elite) and training intensity, but is shorter in over-trained athletes. Similarly, somatic cells are vulnerable to exercise-induced epigenetic modification, including DNA methylation. Exercise-training load, however, depends on intensity and volume (duration and frequency). Training load-dependent responses in genomic profiles could underpin the discordant physiological and physical responses to exercise. In the current review, we will discuss the role of various forms of exercise training in the regulation of DNA damage, TL and DNA methylation status in humans, to provide an update on the influence exercise training has on biological aging.

## Introduction

It is well-established that physical activity and regular exercise can significantly improve overall health and mental wellbeing. Physical activity or exercise can, indeed, reduce the risk of developing several diseases such as obesity, depression, type 2 diabetes, cancers, and cardiovascular disease. Many chronic diseases including obesity (Weiskopf et al., 2009; Ahmad et al., 2012), cardiovascular disease (CVD) (Hansson and Hermansson, 2011; Maynard et al., 2015; Ramos et al., 2016; Sawyer et al., 2016), cancer (Wentzensen et al., 2011; Zhang et al., 2017), psychiatric and neurodegenerative diseases (von Zglinicki et al., 2001; Forero et al., 2016) have been associated with epigenetic changes including DNA damage (Fisher-Wellman and Bloomer, 2009), aberrant DNA methylation profiles (Barres et al., 2012), and short telomeres (Werner et al., 2019). DNA methylation and telomere length can serve as biomarkers of biological age and as predictors of non-communicable diseases (Adwan-Shekhidem and Atzmon, 2018). Numerous coronary artery disease (CAD) risk factors and, particularly type 2 diabetes, are linked to shorter telomeres and cellular senescence (Khan et al., 2012; Zsurka et al., 2018). Additionally, DNA methylation changes at specific genomic locations have been correlated with cancer risk (Huang et al., 2003), diabetes (Liu and Liu, 2010), CVDs (Hegele and Dichgans, 2009), major depressive disorder (Lisoway et al., 2018), autoimmunity disorders (Lu et al., 2006), and aging (Hannum et al., 2013).

Exercise training prevents and manages age-related cardio-metabolic diseases possibly through the regulation of telomere maintenance (Collins et al., 2003; Denham et al., 2013, 2016a, 2016b, 2016c). Alternatively, exercise training could prevent disease through a reduction in disease-related risk factors (blood pressure, lipid profile, adiposity, etc.) (Cassidy et al., 2017), systemic inflammation and oxidative stress (Floyd et al., 1999; Kaszubowska, 2008; Radak et al., 2008; Calle and Fernandez, 2010; Chilton et al., 2014; Steckling et al., 2016), or psychological stress to ultimately prevent telomere shortening. Additionally, short telomeres are associated with reductions in bone mineral concentration in humans (Valdes et al., 2007), muscle cell senescence, apoptosis, or oncogenic transformation of somatic cells, affecting the physical condition, and overall health (Shammas, 2011). Critically short telomeres induce cellular senescence in vascular smooth muscle cells (Uryga and Bennett, 2016), which could also be prevented by exercise training. Several studies have demonstrated that muscle strength and function is related to telomere length in both peripheral leukocytes and skeletal muscle cells, in athletic and apparently healthy populations (Collins et al., 2003; Kadi et al., 2008; Bunout et al., 2009; Rae et al., 2010; Gardner et al., 2013).

It has been shown that cardiovascular disease may be treated and prevented by telomerase reactivation therapies or lifestyle strategies that up-regulate telomerase activity. For instance, Arsenis et al. (2017) revealed that cardiac specific *TERT* gene therapy administered after myocardial infarction enhances cardiac function, telomere extension and lifespan of mice. *TERT* expression is epigenetically controlled through mechanisms such as DNA and histone methylation and histone acetylation (Lewis and Tollefsbol, 2016). Therefore, the combination of exercise training and cardiac medications may improve cardiac rehabilitation and prevent heart failure in the future (Dizon et al., 2013).

Interestingly, endurance athletes with superior cardiorespiratory fitness exhibit twofold and 1.3-fold higher leukocyte *TERT* and *TPP1* gene expression, respectively, relative to their apparently healthy peers (Denham et al., 2016a, b, c). Endurance athletes also possess higher telomerase activity in circulating monocytes (Werner et al., 2008). Therefore, regular aerobic exercise training may facilitate the telomere length maintenance (Garland et al., 2014; Voisin et al., 2015) through a TERT/telomerase mediated process. Understanding the physiological adaptations leading to improved cardiovascular health will allow the development of highly tailored exercise training methods to combat premature biological aging. In fact, according to Recchioni et al. (2017), the inclusion of endurance and/or resistance training into daily life activity appears to alter several miRNAs isolated from peripheral blood (e.g., plasma) and tissues. It is also important to note that some miRNAs may be used as biomarkers of cardiorespiratory fitness (Polakovičová et al., 2016; Denham, 2018). Others have highlighted links between DNA methylation patterns, telomeres length (TL) and exercise training including aerobic or resistance exercise (Dong et al., 2017; Seaborne et al., 2018a, b). However, few studies have investigated the effect of intense training such as sprint training on epigenetic modifications or other structural DNA modifications (TL or DNA damage). In the current review, we will discuss the training modality effect on DNA damage, telomere length and DNA methylation degree across all categories of age. Whilst there are many epigenetic modifications, we focused our attention on the mostly widely investigated structural DNA changes modifying gene expression through the regulation of DNA methylation, TL, and DNA damage.

Information from this review will facilitate the identification of mechanisms of genetic adaptation elicited by acute exercise and the discovery of potential epigenetic adaptations to exercise training that could be utilized in therapeutic interventions for aging and aging-related disease.

## Effect of Acute Exercise on TL, Epigenetic Modifications, and DNA Damage

**FIGURE 1:**
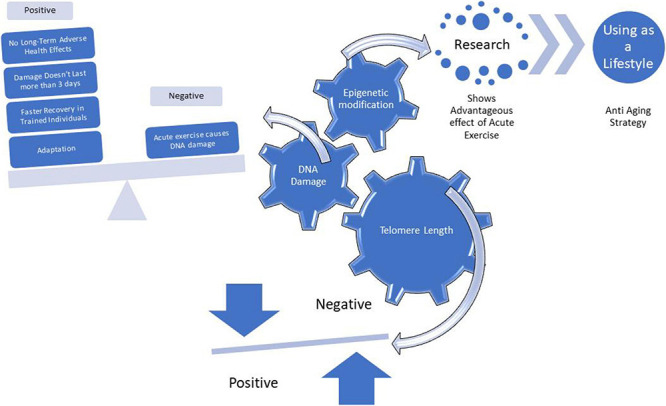
An overview of effects of Acute Exercise on Telomere Length, DNA Damage and Epigenetic modification.

Telomeres are evolutionary conserved DNA found at the ends of eukaryotic chromosomes (in vertebrates, 5*′-TTAGGG^*n*^-3′*) (Greider, 1996; Cawthon, 2002; Blackburn and Epel, 2012). Telomeres preserve genetic information and guard against genomic instability (Allshire et al., 1989; Allsopp et al., 1992; Armanios and Blackburn, 2013). However, with natural aging telomeric DNA is lost with each round of cell division until critical length (Bodnar et al., 1998; Blackburn, 2005; Oeseburg et al., 2010) at which cellular senescence ensues (Effros et al., 2005; Passos et al., 2007; Eisenberg, 2011; Chakarov et al., 2014; Robinson et al., 2018). Given the myriad of lifestyle and environmental factors associated with short telomeres, telomere length is considered as a cellular marker of health status as well as biological aging (Harley et al., 1990; Kappei and Londoño-Vallejo, 2008; Horvath and Raj, 2018; Figure 1).

Different modes of acute exercises might be able to affect the TL and increase the health status and lifespan of a person (Ludlow et al., 2008; Borghini et al., 2015). However, to date, there is a lack of data on the possible acute effects of exercise on TL. Werner et al. (2019) analyzed 124 healthy but inactive individuals for a period of 6 months using continues aerobic endurance training, high-intensive training, and resistance training. It was found that a single bout of endurance training increased telomerase activity in CD14+ and in CD34+ leukocytes. Conversely, no marked changes were observed after acute resistance training. Moreover, Simpson et al. (2010) noticed an immediate increase in TL after acute aerobic exercise session (1 h). Ludlow et al. (2017) reported similar effects in response to acute exercise: an early upregulation of telomere-protective genes as an adaptive mechanism that might contribute to TL maintenance. On the other hand, Mastaloudis reported that exposure to acute endurance exercise might be threatening since it leads to increase the generation of oxygen free-radicals and oxidative stress (Mastaloudis et al., 2001, 2004). Acute exposure to long distance running was also found to cause a decrease in TL due to oxidative DNA damage (Borghini et al., 2015). Further research is necessary to determine the effects of acute exercise (aerobic and resistance training) on TL, telomerase activity and other telomere-regulating molecules (e.g., shelterin and miRNAs) to establish whether telomeres are damaged by acute exercise or if they are rapidly maintained through telomerase-mediated processes. A better understanding of the effect of acute exercise on TL might lead to further utilization of the exercise as an anti-aging lifestyle strategy.

Epigenetics is the study of changes in gene expression that occur in the absence of genetic alterations. Epigenetic modification due to acute exercise could be explained as a structural adaptations similar to the increased transcription of key metabolic, regulatory, and myogenic genes, that are important in mediating subsequent physiological adaptations in skeletal muscle (McGee and Hargreaves, 2019). Barres et al. (2012) showed that a dynamic change in DNA methylation might be responsible for gene activation in skeletal muscle and therefore suggested that DNA hypomethylation might be an early event in muscle contraction-induced gene activation. Grazioli et al. (2017) stated that acute exercise might be able to modulate gene expression by epigenetic alternations, which may lead to potential health benefits. McGee and Hargreaves (2019) also suggested several advantageous healthy benefits achieved through the regulation of genes, mainly controlling muscle structure and function, might be triggered by acute exercise. Furthermore, acute exercise generally induces DNA demethylation at gene promoters. DNA methylation is a form of epigenetic modifications that affects transcriptional activation by allowing the involvement of the transcription machinery to gene promoters. As for other epigenetic modifications, DNA methylation can regulate gene expression following acute exercise without changes to the genetic code (Issa et al., 1994; Seaborne et al., 2018a, b). Fabre et al. (2018), studied the transcriptomic and epigenetic responses after acute exercise in human adipose tissue. It was observed that in relatively similar intensities the magnitude of transcriptomic changes caused by acute exercise was less after endurance training. Fabre et al. (2018), also suggested that training status of individuals differentially affects the epigenetic and transcriptomic responses in human adipose tissue that occur after a single bout of exercise. For example, Jacques et al. (2019), in a recent review emphasized that the field of exercise epigenetics is a relatively youthful field of research and although epigenetic factors are potential biomarkers that could predict the response to acute exercise training, experimental evidence is lacking. Henriksen (2002), stated that a single bout of aerobic exercise lasting 30–60 min at 60–70% of V0_2_ might be able to lower plasma glucose levels significantly. Although researchers mentioned numerous beneficial effects of acute exercise on epigenetic modifications, it is still unclear.

Extensive research has been conducted in the domain of acute exercise and DNA damage in the past few decades. Along with research for healthy populations, the effect of acute exercise on DNA damage has been studied among individuals with cardiovascular disease (Sahlin et al., 1991; Jimenez et al., 2000; Fisher-Wellman and Bloomer, 2009), hypercholesterolemia (Chen et al., 1994; Fisher-Wellman and Bloomer, 2009), diabetes (Laaksonen et al., 1996; Davison et al., 2002; Villa-Caballero et al., 2007; Fisher-Wellman and Bloomer, 2009), obesity (Vincent et al., 2004, 2005), chronic obstructive pulmonary disease (Heunks et al., 1999; Fisher-Wellman and Bloomer, 2009) and intermittent claudication (Silvestro et al., 2002; Fisher-Wellman and Bloomer, 2009). Fisher-Wellman and Bloomer (2009), in the review of last 30 years research on the topic stated that both acute aerobic and acute anaerobic exercise increase the production of reactive oxygen and nitrogen species (RONS) resulting in oxidative stress in both human and animal models. DNA damage due to oxidative stress has been considered in the literature as a negative factor for health. Belviranlı and Gökbel (2006) deemed the increased generation of oxidants with acute exercise as a normal physiological response to acute exercise. Belviranlı and Gökbel (2006) further suggested that acute exercise incorporated as a lifestyle change might play a vital role in preventing oxidant production by increasing the activity of antioxidant enzymes. Moreover, DNA damage seems to be significant only up to 1-day post-acute aerobic exercise and seems to reduce after 5–28 days post exercise. Tryfidou et al. (2020), in a recent review of DNA damage caused by acute aerobic exercise, suggested that the development of a multi-dimensional model for a better understanding of the complex process of acute exercise and DNA damage should be considered. The exercise-induced DNA damage should not necessarily be considered as a harmful consequence since it is most likely repaired within the following 3 days (Belviranlı and Gökbel, 2006; Fisher-Wellman and Bloomer, 2009; Tryfidou et al., 2020). No long-term adverse health outcomes of acute exercise have been reported in the literature (Reichhold et al., 2008). Many factors might be responsible in contributing to the DNA damage during acute exercise, such as the mode of exercise, duration of the exercise, and intensity of exercise. Training status (Reichhold et al., 2008) and dietary intake (Belviranlı and Gökbel, 2006) differences amongst individuals might also regulate the extent to which DNA damage occurs following exercise. Training status (e.g., high cardiorespiratory fitness) also appears to be a vital factor when it comes to adaptation. Acute exercise seems to protect immune cells against radiation-induced DNA strand breaks in trained subjects (Moreno-Villanueva et al., 2019). Therefore, it can be said that incorporating acute exercise, especially acute aerobic exercise as a healthy aging strategy might be beneficial.

## Exercise Training-Induced Epigenetic Reprogramming

Much of the decrease in cardiorespiratory fitness and muscular function of the elderly is related to the decline in physical activity. Indeed, physical inactivity in a major contributor to aging (Stewart, 2005). For example, the decrease in aerobic capacity is approximately 10% per decade, partly related to the environmental and lifestyle changes (Fleg et al., 2005; Milanović et al., 2013). In patients with chronic disease, the loss of these abilities is augmented. The decrease in strength and muscle mass with age (sarcopenia) is mainly related with the decrease in physical activity with age, but also with nutritional and hormonal factors (Baumgartner et al., 1998; Thomas, 2007). However, it is possible to improve or maintain aerobic capacity and muscular strength through regular physical training up until advanced age (Sellami et al., 2014). Recent evidence suggests that exercise training may prevent accelerated biological aging and maintain physical performance through epigenetic reprogramming.

Zhang et al. (2011) found a positive association between physical activity and global DNA methylation in 45- to 75-year-old Hispanic subjects. Typically, global DNA methylation is lower in older patient than younger groups (Fraga et al., 2005). Regular exercise training has been associated with alterations in gene expression and methylation of specific genes. For example, lower mRNA expression of toll-like receptor 4 (TLR4) and CD14 was observed shortly after resistance training (Flynn et al., 2003), a higher methylation level was observed for Apoptosis-associated speck-like protein containing a caspase recruitment domain (ASC), an adaptor molecule that mediates inflammatory cytokines IL-1β and IL-18 (Nakajima et al., 2010).

Physical exercise alters gene expression, as cells adapt to the metabolic change during a bout of exercise. Occasional aerobic exercise can also change the methylation profile of DNA (Norrbom et al., 2010, 2011), but if exercise is not regular epigenetic adaptations will regress. There seems to be an intensity-dependent relationship between aerobic exercise and the degree of DNA methylation changes, at least in skeletal muscle. As an example, more marked DNA methylation changes occurred at the promoter region of genes important for skeletal muscle adaptations in muscle from those who complete a single bout of high intensity exercise compared to those who ran at a lower intensity (Barres et al., 2012). As such high-intensity physical activity could activate more genes (Ntanasis-Stathopoulos et al., 2013), but whether the changes are permanent or reversible is unclear.

## Effects of Aerobic Training on DNA Damage, DNA Methylation and Telomere Length

Current evidence suggests regular engagement in exercise training may attenuate telomere attrition, though most is correlative or by association only. Recent evidence suggests that the pace of aging and onset of age-associated cardiovascular diseases (i.e., stroke, heart failure, and atherosclerosis) is affected by TL (Minamino and Komuro, 2007; Arsenis et al., 2017). According to Matthews et al. (2006), shortened TL leads to cellular senescence and subsequently atherosclerosis. In fact, the process of aging and atherogenesis are related to molecular mechanisms, such as an increase in reactive oxygen species (ROS), decreased nitric oxide bioavailability, and reduced telomerase reverse transcriptase activity (Yeh and Wang, 2016).

It will be difficult to isolate the influence that exercise training has on actual telomere length shortening as this process occurs slowly in humans and requires large, long-term studies (over many years). Telomere length may fluctuate over time, but ultimately shortens in the long term. Consequently, the optimal exercise recommendations for TL maintenance remain elusive. LaRocca et al. (2010) found that the leukocyte telomere maintenance was higher in trained compared to untrained adult individuals. Similar results in other athletic populations have supported Laye et al. (2012) and demonstrated that athletes with superior fitness levels typically exhibited longer leukocyte and skeletal muscle telomeres compared to control cohorts (LaRocca et al., 2010; Denham et al., 2013, 2016a, 2016b, 2016c). Ponsot et al. (2008) revealed that the regular physical activity performed by old subjects is not associated with accelerated telomere loss in skeletal muscle tissue. However, numerous studies examining the effect of low to moderate-intensity aerobic training on telomere length were based on questionnaire evaluations of physical activity. Some recent studies have addressed this issue by including an objective test of cardiorespiratory fitness (e.g., a test of maximal oxygen consumption).

Numerous studies have investigated the effect of **low and moderate intensity aerobic exercise** on DNA damage. Niess et al. (1996) found high levels of DNA damage after incremental treadmill test until exhaustion in trained compared to untrained subjects. Interestingly, these changes were less pronounced in the trained groups, and plasma levels of malondialdehyde (MDA) were significantly lower in trained compared to untrained groups, suggesting that regular moderate intensity training could protect against exercise-induced DNA damage.

Werner et al. (2019) found that the endurance training acutely increased telomerase activity and TL. Moreover, Simpson et al. (2010) noticed an immediate increase in TL after acute aerobic exercise session. Ludlow et al. (2017) reported similar effects in response to acute exercise that might contribute in maintaining TL. Acute exposure to long distance running decreased TL, possibly due to oxidative DNA damage (Borghini et al., 2015). Further research is necessary to establish what effects acute exercise has on telomere biology.

### High-Intensity Aerobic Exercise Training, DNA Damage and DNA Methylation

Barres et al. (2012) studied the exercise intensity effect (40% of VO_2max_ vs. 80% of VO_2max_) on DNA methylation level in biopsies of vastus lateralis skeletal muscle and gene activation in young sedentary men and women before and after an acute exercise. Similarly, to Coffey and Hawley (2007) and Pilegaard et al. (2000), Barres and colleagues found that exercise performed until fatigue (maximal progressive cycling test in a fasted condition) decreases promoter methylation of the PGC-1α and PDK4 genes, while no changes are observed in PPAR-γ methylation. Barres et al. (2012) hypothesized that mechanism behind the rapid demethylation may be due to a loss of methyl groups rather than hydroxylation. However, it has been shown that 6-month of vigorous training including one session of 1 h spinning and two sessions of 1-h aerobics resulted in increased methylation in adipose tissue in response to exercise in sedentary middle-age men (Rönn et al., 2013). The researchers also found differential methylation at individual CpG sites ranging from 0.2–10.9%.

Zhang et al. (2011) demonstrated that more vigorous activity (increased volume of physical effort per week, i.e., 26–30 min/day vs. ≤10 min/day) measured by accelerometers resulted in an increase in global genomic DNA methylation of white blood cells in non-Hispanics, despite the interference of gender on the results. Similarly, Nakajima et al. (2010) observed that high-intensity interval walking for 6 months increased methylation degree of *ASC* gene in 230 individuals from experimental group compared to their controls. ASC-mediated inflammation through secretion of pro-inflammatory cytokines IL-1β and IL-18 were also found to play a critical role in tumor development (Deswaerte et al., 2018). According to Poulsen et al. (1996), intense exercise training for ∼10 h a day (including long distance running) over a 1-month period increased the rate of oxidative DNA modification by 33% in young men at a military camp.

Based on several findings from previous research on the effect of a single bout of vigorous acute exercise in trained or untrained subjects (cross sectional studies), it is clear that exercise leads to a high degree of oxidative DNA damage (Poulsen et al., 1996; Møller et al., 2001; Tsai et al., 2001; Reichhold et al., 2009; Çakır-Atabek et al., 2015). In fact, a 42 km marathon race has been found to alter DNA base oxidation in peripheral immunocompetent cells (Tsai et al., 2001). Tryfidou et al. (2020), in a recent review of DNA damage caused by acute aerobic exercise suggested the development of a multi-dimensional model for a better understanding of the complex process of acute exercise induced DNA damage. The DNA damage that occurs as a consequence of acute aerobic training should not necessarily be considered as a negative outcome. As mentioned previously the duration of the negative effects is less than 3 days and no adverse health effects have been mentioned in the literature (Belviranlı and Gökbel, 2006; Fisher-Wellman and Bloomer, 2009; Tryfidou et al., 2020). Therefore, it should be deemed as physiologically normal and an important process responsible for signaling exercise-induced adaptations. However, trained individuals with high cardiorespiratory fitness exhibit a better recovery rate compared to untrained subjects, indicating its importance in adaptive responses., which can be utilized and incorporated as an healthy-aging strategy.

## Effect of Anaerobic Training on DNA Damage, DNA Methylation and Telomere Length

The most widely studied form of training for the anaerobic energy system is running and cycling, which involves very short bouts of vigorous exercise performed at maximum speed—generally seconds up to a few minutes. Interval training has been an effective type of training for decades but recently variations, such as high-intensity interval training, including sprint interval training (SIT), are growing in popularity.

Denham et al. (2014) found marked changes to the leukocyte DNA methylome after a 4-week sprint interval training (SIT) intervention. The DNA methylation changes occurred with concurrent modulation to gene and mature microRNA (e.g., miR-21 and miR-210), and improvements in cardiovascular health and performance in young men after the 4-week SIT—running—intervention. A similar 12 weeks SIT intervention was associated with modest DNA methylation changes in mature sperm of healthy young men at genes implicated in a host of developmental and chronic diseases (Denham et al., 2015a, b). Furthermore, Larsen et al. (2015) showed that sprint training is a powerful stimulus of mitochondrial biogenesis pathways and increases oxidative balance and mitochondrial density.

Intense exercise training seems to lead to marked DNA methylation changes throughout the genome of various somatic cells. In fact, some data on more intensive exercise (i.e., High intensity exercise) reported a reduction in DNA methylation at specific mitochondrial genes (Barres et al., 2012) and genome-wide (Denham et al., 2015a, b), while other studies suggested an overall increase in DNA methylation in adipose tissue in middle-age men (Rönn et al., 2013). It is important to note that changes in CpG methylation should be measured in context with the genomic location and with other epigenetic modifications to gain a better understanding of their function. For instance, an increase in gene body methylation is associated with transcriptional activation, whereas promoter methylation can result to gene inactivation. DNA methylation is typically governed in concert with other epigenetic modifications (e.g., histone acetylation and methylation) and small non-coding RNAs.

Laine et al. (2015) investigated the effect of vigorous elite-class physical activity on leukocyte TL between endurance, power lifters and mixed sport athletes. Missed sports including soccer, ice hockey, basketball, jumpers, sprinters, and hurdlers were mostly activities based on anaerobic exercises. Interestingly, the authors found no significant differences between all athletic groups and controls. The authors explained that exercise training intensity may not influence TL in adulthood. These results are consistent with recent evidence suggesting cardiorespiratory fitness is not associated with leukocyte or skeletal muscle telomeres (Hiam et al., 2020). However, few studies report the inverted U relationship (Ludlow et al., 2008; Savela et al., 2013) between chronic exercise and TL and the majority of positive findings indicating longer leukocyte telomeres in physically active and cardiorespiratory fit individuals (LaRocca et al., 2010; Krauss et al., 2011; Denham et al., 2013, 2016a, 2016b, 2016c; Mason et al., 2013). Notably, the influence of exercise on telomere length appears to be cell-type specific. Telomere length is preserved in the heart and liver but is shorter in the skeletal muscle of exercised mice, compared to sedentary controls (Ludlow et al., 2013). Endurance exercise was associated with longer whole blood leukocytes (Denham et al., 2013) but not in isolated PBMCs (Denham et al., 2016a, b, c). Similar findings have also suggested more marked effects of endurance exercise on granulocytes compared to whole blood leukocytes (Werner et al., 2008). These findings indicate possible cell-specific effects of exercise that should be investigated in future work.

There are currently limited investigations on influence of intensive sports with anaerobic system as primarily source of energy and their relationship with TL. Recently, Simoes et al. (2017) investigated the TL as well as body composition and aspects of athletic performance in elite sprinters. Master sprinter athletes who practiced intensive training for 10 years and competed in 60–400-meter dash or hurdle events were compared to non-trained athletes. The DNA was extracted from peripheral blood mononuclear cells (PBMC). In this study, elite sprinters possessed longer telomeres, healthy lipid and body composition profiles and higher sprint performances (in terms of % of world record) compared to the age- and sex-matched control group. The researchers suggested that a longer TL is positively correlated with fitness levels. However, the limitations of the study are methodological limitations that require larger sample size that would give more meaningful results, both for the athletic and control groups, respectively. The basic mechanism of TL preservation in elite sprinters remains to be experimentally confirmed in future studies.

## Effect of Resistance Training on DNA Damage, DNA Methylation and Telomere Length

Resistance training includes all types of exercises that cause muscles to contract against an external resistance, which increases muscular strength, tone, mass, and/or endurance. While most studies have investigated the relationship between aerobic exercise training and TL, few studies have investigated the relationship between resistance training and DNA damage or TL. Regarding the former, most studied the effects of acute exercise (Çakır-Atabek et al., 2015).

According to Seaborne et al. (2018a, b) skeletal muscle *GRIK2*, *TRAF1*, *BICC1*, and *STAG1* DNA are vulnerable to DNA methylation changes elicited by a single bout of resistance exercise and these effects are maintained 22 weeks after the exercise session in adult male. Resistance training also modulate the leukocyte DNA methylome and transcriptome in genes responsible for numerous pathways including growth factors, in healthy young men (Denham et al., 2016a, b, c). However, resistance training does not appear to induce as many DNA methylation and transcriptional changes compared to those induced by aerobic exercise training. Robinson et al. (2017) found that both 12-week of resistance training and HIIT allow small changes of methylation of DNA promoter regions.

Compared to aerobic exercise training the available literature on the effects of resistance training on telomere length, DNA methylation and DNA damage are limited. For example, Flynn et al. (2003) observed lower mRNA expression of toll-like receptor 4 (TLR4) and CD 14 following resistance training in elderly women. Kadi et al. (2008) investigated the effect of regular resistance training on TL among power lifters athletes and young healthy controls. No marked differences in skeletal muscle minimum and mean TL were observed in trained power lifters compared to untrained controls. They did, however, reveal an inverse correlation between personal best strength achievements and TL. Although considered a safe an effective intervention for improving quality of life and muscular strength, Hagstrom and Denham (2018) found that a 16-week resistance training intervention did not alter leukocyte TL in women recovering from breast cancer compared to a control group. Given that telomeres gradually shorten with aging, longer interventions will be required to investigate any meaningful exercise-induced changes in TL in longitudinal studies.

Melov et al. (2007) also investigated the effects 6-month moderate to intense resistance training in young and older adult. Training progressed from exercise at 50% of the initial 1 repetition maximum (RM) to three sets at 80% of 1RM by the end of the experimental period. A muscle biopsy of vastus lateralis muscle was performed, and RNA was extracted to evaluate the transcriptome profile. Interestingly, they found that this type of training helped reverse the transcriptional signature of older individuals to that of younger in most genes affected by both age and exercise.

Murphy et al. (2008) found that 12 weeks of resistance training performed at moderate intensity improved muscle oxidative capacity, myofibril damage and regeneration and higher fraction of neural cell adhesion molecule (NCAM)-positive satellite cells in eight patients with single, large-scale sporadic mitochondrial DNA (mtDNA) deletions. In the same context, it has been demonstrated that resistance exercise training was advised to patients with mitochondrial disease, referred to as “gene shifting” (Johnston et al., 2007).

Dimauro et al. (2016) investigated the effect of 12-week of low frequency, moderate intensity, explosive-type resistance training (3–4 sets of 10–12 repetitions at 70% of 1RM) on TL, global DNA methylation, TRF2, Ku80, SIRT1, SIRT2 and global protein acetylation, proteins such as Bcl-2, Bax and Caspase-3 and oxidative status in 72-year-old individuals. DNA was analyzed using whole blood. Relative to the control group, those who completed the resistance training exhibited longer TL after the intervention period. Although they found no changes in TRF2, Ku80 protein expression among groups, there were significant lower protein expression of antioxidant proteins in PBMCs (MnSOD, TrxR1 and serum MPO) in the trained group following the intervention. Interestingly in this study, the global DNA methylation level was lower in the trained group compared to control cohort, with no changes of SIRT1 and SIRT2 content. Dimauro et al. (2016) suggested that TL shortening is linked to changes in redox homeostasis markers.

Soares et al. (2015) investigated the effect of 16-week of combined strength and aerobic training including session of moderate intensity aerobic exercises at 55–75% of heart rate reserve (walking, running, biking, rowing, and elliptical) and moderate intensity strength exercises at 55–75% of 1RM (2–3 sets of 10–15 repetitions) in 50 healthy adult (40 years) and older (70 years) subjects. To measure DNA damage, authors used the DNA strand breaks (DNA SBs) and oxidative DNA damage (FPG-sensitive sites). Interestingly, the intense training and in combination with aerobic exercise attenuated DNA SBs, FPG-sensitive sites, and Malondialdehyde (MDA) levels, without changes in OGG1 activity, the main enzyme regulating the excision of 8-oxoguanine (8-oxoG). Such results were assumed to be due to reductions in oxidative stress levels through decreased ROS and lower production of oxidants. Their results were supported by higher total antioxidant capacity in trained individuals compared to their age-matched control group.

In another study, Franzke et al. (2014) study the effect of 6-month of strength or resistance training (low to heavy intensity) combined with or without a diet strategy based on supplementation of protein and vitamin, on DNA strand breaks in old (65 years) and elderly (98 years) men and women. To assess the DNA damage, they measured the net amount of FPG-sensitive sites and for oxidative stress they calculated the superoxide dismutase (SOD), the catalase (CAT) activity and Glutathione peroxidase (GSH-Px). They identified an increased rate of DNA damage in all groups despite their different genders, yet the increased rate was within normal ranges when compared to previous studies.

Recently, Gargallo et al. (2018) investigated the effect of 16-week progressive resistance training program with elastic bands performed at a moderate (15-submaximal repetitions at 70% of 1RM) and high intensity intensities (6-submaximal repetitions at 85% of the 1RM) on systemic redox state, DNA damage, and fitness level of sedentary older women (age range 60–75 y). The 8-OHdG assay in urine by high-pressure liquid chromatography-electrochemical detection (HPLC-EC) was used to determine DNA oxidation. The research group found that more intense resistance training resulted in increases of oxidative stress expressed by higher urine 8-OHdG levels and lower antioxidant tripeptide GSH, while moderate training allow reduction in DNA damage with no alteration in GSH or the GSSG/GSH ratio despite improvement in physical performances in both groups.

It is reasonable to investigate the role of hormones, particularly those involved in anabolic processes, to explain adaptations to resistance training. Hormones play a key role in regulating telomere damage. In fact, Bayne and Liu (2005) explained in their review how growth hormones and growth factors are involved in telomere length regulation. They concluded that the estrogen upregulates *hTERT* gene expression and telomerase activity in several estrogen receptor (ER) of positive cancer cell lines, while androgen increased this activity in prostate cancer cells but reduced it in normal prostate. Sex hormones seem to facilitate telomere elongation *in vivo*, as the administration of the danazol for 24 months leads to telomere elongation in patients with various telomere-related diseases (Townsley et al., 2016). Numerous other studies found also that while TGF-β reduces telomerase activity (Katakura et al., 1999; Yang et al., 2001), EGF, IGF-1 and IGFBP-3 increase its activity (Maida et al., 2002; Budiyanto et al., 2003; Moore et al., 2003; Wetterau et al., 2003). Resistance training has been shown to alter secretion of growth factors and growth hormones by reducing the aging effect of these hormones (Sellami et al., 2017). Epel (2009) suggested that reduction of anabolic hormones such as testosterone, growth hormones and IGF-1 is correlated with reduction of TL, but with resistance training, the anabolic hormones seem to be enhanced, which may be favorable to counteract the negative effects of age on TL. Indeed, mice treated with either IGF-1 or growth hormone exhibited up-regulated myocardial telomerase activity (14-fold and eightfold, respectively) (Werner et al., 2008). In addition, a reduction in oxidative stress has also been demonstrated following resistance training, which could enhance telomerase activity (Fitzpatrick, 2006; Fitzpatrick et al., 2007). Furthermore, shorter TL is linked to higher adiposity (Gardner et al., 2005) and resistance training has been shown to improve body weight and composition in young and adult subjects.

## Discussion and Summary

There appears to be inconsistencies amongst investigations studying the effect of exercise training on TL: positive relationships, no statistically significant association and inverted U-shaped relationships have been described (Du et al., 2012; Savela et al., 2013; Denham et al., 2014). The lack of consistent findings are possibly due to differences in ethnic/genetic diversity of subjects between studies, study populations’ age range, method of DNA extraction and the type of cell analyzed, but mainly the different type of exercise training (intensity and volume) and its quantification.

The consensus from the above studies is that, in sedentary individuals TL decreases with age, yet in individuals who are moderately active longer telomeres are observed. However, extreme long-duration endurance training for an extended portion of one’s lifetime may result in telomere shortening, at least in skeletal muscle. Endurance exercise does not appear to be detrimental to leukocyte or muscle TL maintenance, according to the current data on endurance exercise and resistance training. There could, however, be an upper limit to the protective effect of exercise depending on volume (duration and frequency) and intensity of training, considering shorter muscle telomeres were inversely correlated to maximal strength and training history (years and hours spent training) (Collins et al., 2003).

## Conclusion

Chronic exercise is an inexpensive activity that plays important roles in telomere maintenance and DNA methylation levels, possibly through its ability to lower oxidative stress and inflammation. Most studies have shown that regular physical activity increases the activity of telomerase and attenuates telomere shortening in human leukocytes. The current review demonstrated that aerobic exercise training and/or endurance training appears the most effective in conserving telomere length when compared to other type of training based on anaerobic exercises. In addition, the low volume with moderate intensity resistance training appears beneficial to maintain the TL in older adult, though longitudinal studies are required to confirm these assertions.

Although sprint-training causes marked changes in the leukocyte DNA methylome, results of the effect of sprint training are still scarce in comparison to other exercise interventions and do not currently support the “anti-aging” effects. As such, further understanding of global DNA methylation, oxidative damage, and telomere length would shed light on the role of epigenetic modifications at play in physical conditioning and may ultimately support the development of effective therapy to counteract the deleterious effects of aging.

## Author Contributions

MS and NB conceived the review and drafted the manuscript. MS, NB, MP, JD, and ME revised the manuscript. All authors contributed to the article and approved the submitted version.

## Conflict of Interest

The authors declare that the research was conducted in the absence of any commercial or financial relationships that could be construed as a potential conflict of interest.

## Publisher’s Note

All claims expressed in this article are solely those of the authors and do not necessarily represent those of their affiliated organizations, or those of the publisher, the editors and the reviewers. Any product that may be evaluated in this article, or claim that may be made by its manufacturer, is not guaranteed or endorsed by the publisher.
